# Anthocyanins from purple corn activate free fatty acid-receptor 1 and glucokinase enhancing *in vitro* insulin secretion and hepatic glucose uptake

**DOI:** 10.1371/journal.pone.0200449

**Published:** 2018-07-11

**Authors:** Diego A. Luna-Vital, Elvira Gonzalez de Mejia

**Affiliations:** Department of Food Science and Human Nutrition, University of Illinois at Urbana-Champaign, Urbana, Illinois, United States of America; Medical University of Vienna, AUSTRIA

## Abstract

The objective of this study was to evaluate the ability of anthocyanins (ANC) present in purple corn to enhance insulin secretion and hepatic glucose uptake in pancreatic cells and hepatocytes, through activation of the free fatty acid receptor-1 (FFAR1) and glucokinase (GK), respectively. Using a dual-layer cell culture with Caco-2 cells, INS-1E or HepG2 cells were treated with an anthocyanin-rich extract from the pericarp of purple corn (PCW), as well as pure ANC cyanidin-3-*O*-glucoside (C3G), peonidin-3-*O*-glucoside, pelargonidin-3-*O*-glucoside. Delphinidin-3-*O*-glucoside (D3G) was used for comparative purposes. Semipurified C3G (C3G-P) and condensed forms (CF-P) isolated from PCW were also used. At 100 μM, the pure ANC enhanced glucose-stimulated insulin secretion (GSIS) in INS-1E cells ranging from 18% to 40% (p<0.05) compared to untreated cells. PCW increased GSIS by 51%. D3G was the most effective anthocyanin activating FFAR1 (EC_50_: 196.6 μM). PCW had activating potential on FFAR1 (EC50: 77 μg/mL). PCW, as well as C3G and D3G increased the expression of FFAR1, PLC, and phosphorylation of PKD, related to the FFAR1-dependent insulin secretory pathway. The treatment with 100 μM of P3G and C3G increased (p<0.05) glucose uptake in HepG2 cells by 19% and 31%. PCW increased the glucose uptake in HepG2 cells by 48%. It was determined that CF-P was the most effective for activating GK (EC_50_: 39.9 μM) and the PCW extracts had an efficacy of EC_50_: 44 μg/mL. The ANC in purple corn also reduced AMPK phosphorylation and PEPCK expression in HepG2 cells, known to be related to reduction in gluconeogenesis. It is demonstrated for the first time that dietary ANC can enhance the activity of novel biomarkers FFAR1 and GK and potentially ameliorate type-2 diabetes comorbidities.

## Introduction

Diabetes is a complex metabolic disease that affects more than 30 million people in the United States (U.S.) and it has been estimated that this number will become approximately 48.3 million in 2050 [1, 2]. The inability to control blood glucose increases the risk of severe health complications and thus diabetes remains as a leading cause of death in the U.S. [2]. Type-2 diabetes, which accounts for 90–95% of total diabetes cases, is characterized by an increased resistance to insulin action and/or deficiency in insulin secretion [3]. Although the exact etiology for this condition is unclear, epidemiologic studies have shown that older age, poor dietary habits, physical inactivity and excess body weight are some of the main risk factors [4–6]. Dietary intervention strategies have been shown to be beneficial to control hyperglycemia, especially the intake of whole grains, fruits, vegetables, nuts and legumes [7]. The benefits associated with a healthy diet could be related to the presence of dietary components, such as unsaturated fatty acids, dietary fibers and certain phytochemicals. Therefore, considerable attention has been given to developing food ingredients that are promising to prevent and manage diabetes. For example, the increased consumption of polyphenols, such as anthocyanins (ANC), has been correlated with a lower incidence of type-2 diabetes in humans [8–10].

ANC are naturally occurring pigments widely spread in vivid colored fruits, vegetables and grains. They commonly exist as glycosides of flavylium (2-phenylbenzopyrylium) salts and are mainly based on six anthocyanidins: pelargonidin, cyanidin, peonidin, delphinidin, petunidin and malvidin [11]. With regard to their chemical nature, ANC differ in hydroxyl substitution patterns, degree of methylation, the nature and position of sugar moieties, and the presence of aliphatic or aromatic acids [12]. This diversity in structures allows ANC to interact differently with other molecules and therefore, endow the compounds with unique biological properties. Recently, several *in vitro* and *in vivo* studies have revealed the anti-diabetic potential of ANC [13]. It was shown that a mulberry ANC extract can enhance glucose consumption, suppress hepatic gluconeogenesis and modulates glucose metabolism in human HepG2 cells [14]. Another study reported that a C3G-enriched extract from blood orange juice and its secondary metabolite can improve glucose tolerance and insulin sensitivity in diet-induced obese mice [15]. Other ANC-rich extracts from different sources such as blackcurrant [16], red raspberry [17], purple rice [18] and black soybean [19] have also been demonstrated to possess hypoglycemic abilities. Among them, the ANC from colored corn, especially purple corn, has shown promising anti-diabetic activities [13]. Our previous study found that an ANC-rich extract from purple corn pericarp ameliorated insulin resistance and enhanced glucose uptake in 3T3-L1 adipocytes [20].

Recently, free fatty acid receptor 1 (FFAR1, also known as GPR40) and glucokinase (GK) have attracted high interest as two novel targets for type-2 diabetes [21, 22]. FFAR1, also known as GPR40, is a receptor to medium-to-long chain fatty acids and can stimulate glucose-dependent insulin secretion in pancreatic β-cells [23]. It has been found that the activation of FFAR1 ameliorates the fasting hyperglycemia and glucose tolerance in diabetic rats [24]. More importantly, no hypoglycemic effects and pancreatic toxicity were observed in normal rats, suggesting selective beneficial effects for type-2 diabetes patients [24, 25]. GK, primarily expressed in pancreatic β-cells and liver hepatocytes, is known to play a crucial role in glucose homeostasis [26]. In the case of insulin resistance and type-2 diabetes, there was a significant lower level of hepatic GK expression, suggesting the underlying dysregulation of this biomarker [27]. After GK activators action, decreased blood glucose levels in diet-induced obesity mice were observed [28, 29]. More recently, several studies have reported the impacts of polyphenols on these two novel diabetes targets [30–33]. However, there is no information about the interaction of ANC from dietary sources with FFAR1 or GK and their relationship with insulin secretion and glucose uptake. Therefore, the objective of this study was to evaluate the potential of ANC extracted from purple corn, as well as pure ANC cyanidin-3-*O*-glucoside (C3G), peonidin-3-*O*-glucoside (P3G), pelargonidin-3-*O*-glucoside (Pr3G) and delphinidin-3-*O*-glucoside (D3G), to activate FFAR1 and GK, and consequently to increase insulin secretion and hepatic glucose uptake *in vitro*.

## Materials and methods

### Materials

Commercially available purple corn was purchased from Angelina’s Gourmet (Lot No. L670106, Swanson, CT). Caco-2 cells and HepG2 cells were obtained from ATCC (Manassas, VA). iNS-1E cells were kindly provided by Dr. Maechler [34]. Pure ANC (purity ≥ 96%) C3G, P3G, Pr3G, and D3G were purchased from Extrasynthese (Genay, France). FFAR1 agonist Fasiglifam (TAK-875), antagonist GW-1100, and GK activator Ro-28-1675 were purchased from MedChem Express (Monmouth Junction, NJ). Primary rabbit polyclonal antibody FFAR1 (rabbit polyclonal Cat. No. ab211049) was obtained from Abcam (Cambridge, MA). Primary antibodies AMPK (rabbit monoclonal Cat. No. sc-25792), PEPCK (mouse monoclonal Cat. No. sc-271029), and GAPDH (mouse monoclonal Cat. No. sc-32233) were purchased from Santa Cruz Biotechnology (Santa Cruz, CA). Primary antibodies PLC (rabbit polyclonal Cat. No. 2822), PKD (rabbit monoclonal Cat. No. 90039), p-PKD (Ser744/748 rabbit monoclonal Cat. No. 2054), and p-AMPK (Thr172 rabbit monoclonal Cat. No. 2535) were purchased from Cell Signaling (Danvers, MA). All other reagents were purchased from Sigma-Aldrich (St Louis, MO) unless otherwise indicated.

### Anthocyanin-rich extracts preparation

Purple corn pericarp was extracted using a dry milling procedure [35]. An ASE 300 Accelerated Solvent Extraction System (Thermo Scientific) was used to extract anthocyanin from purple corn pericarp, using only water as solvent. Corn pericarp (about 10 g) was added to a stainless steel cell with cellulose filter. The cell was preheated for 5 min and heated up to 50°C for extraction; static solvent extraction time was 5 min. The pressure in the cell during extraction was 1500 psi. The amount of water to flush through the cell following the static heating step was 100% of the cell volume. Extraction cycle to perform the static heating and flushing steps was five times. Cells were purged with nitrogen for 60 s at the end of the extraction. After freeze-drying, the extracts were passed through a Sephadex® LH-20 resin column (25 x 1.7 cm) using only water as eluent in an AKTAprime plus chromatography system (GE Healthcare, Milwaukee, WI). The volume of the water extracts was reduced by 60% in a rotational evaporator, freeze-dried, and kept at -20°C until further use. The resulting powder was called purple corn anthocyanin-rich water extract (PCW).

### Anthocyanin quantification by HPLC

HPLC analysis of samples for ANC profile was performed in triplicate using a Hitachi HPLC System (Hitachi High Technologies America, Inc., Schaumburg IL) equipped with a diode array multi-wavelength detector, L-7100 pump following the previously reported protocol [36] with some modifications. The injection volume was 20 μL. The flow rate was 1 mL/min and the gradient used was from 2% formic acid in water and 0% acetonitrile to 40% acetonitrile in a linear fashion using a Grace Prevail C18 (5 μm, 250 × 4.6 mm, Columbia, MD) for 30 min. Peaks were identified based on the same retention time as available standards or literature for the condensed forms. The concentration of each ANC was determined using a calibration curve with 5 points in the range of 50–2000 ng/ml of the different standards. The calibration curve was obtained by plotting the area of ANC peak versus concentration at 520 nm. The calibration curve requires a correlation coefficient (*r*^2^) of 0.99 or better. The results were expressed as mg/g dry weight.

### Isolation and identification of anthocyanins from anthocyanin-rich extracts

After ANC processing, the extract from purple corn was passed through an Amberlite® XAD7HP resin column in an AKTA prime plus chromatography system (GE Healthcare, Milwaukee, WI) eluting with 30% aqueous ethanol. Different fractions were collected from the 280 nm chromatogram. They were analyzed using previously reported conditions [36] in a Hitachi HPLC System (Hitachi High Technologies America, Inc., Schaumburg IL) equipped with a diode array multi-wavelength detector. A Grace Prevail C18 (5 μm, 250 × 4.6 mm, Columbia, MD) column was used to compare with pure standards. The resulting fractions were called semi-purified cyanidin-3-*O*-glucoside from purple corn (C3G-P), and semi-purified condensed forms from purple corn (CF-P). C3G-P and CF-P were analyzed by tandem mass spectrometry using conditions previously described [36] on a Waters quadrupole time-of-flight (Q-Tof) Ultima equipped with an electrospray ionization (ESI) interface and controlled by MassLynx V4.1 software (Waters Corp., Milford, MA, USA). A Sunfire® C18 column (150 mm × 2.10 mm, 3.5 μm, Waters Corp., Milford, MA, USA) was utilized for separation. According to the MS^2^ analysis, the molecular mass of the condensed forms was 899 g/mol. Such mass value was used to calculate the concentration of CF-P in molarity units.

### Cell culture and dual-layered cell culture system

Caco-2 cells were subcultured using MEM supplemented with 20% FBS, 1% penicillin–streptomycin, and 1% sodium pyruvate at 37°C in a humidified atmosphere with 5% CO_2_. HepG2 cells were maintained in DMEM supplemented with 10% FBS and 1% antibiotic. iNS-1E cells were maintained in RPMI-1640 medium containing 10 mM HEPES, 2 mM l-glutamine, 1 mM sodium pyruvate, 50 μM 2-mercaptoethanol, and 4500 mg/L glucose, supplemented with 10% FBS and 1% amphotericin B. A dual-layered cell culture system was used. The different treatments were applied to the apical side of human epithelial Caco-2 cells grown in monolayer inserts above iNS-1E pancreatic β-cells or HepG2 hepatic cells to determine the biological effect of transported ANC, or their metabolites. Hanging cell culture inserts contained a polystyrene membrane with a 0.4 μM pore that according to previous research, allows transport of phenolic compounds [37]. Caco-2 cells were seeded on the inserts, at a density of 5 × 10^4^ cells/cm^2^ to start an intestinal epithelial monolayer. Integrity of Caco-2 cells monolayer was evaluated by transepithelial electrical resistance (TEER) measurements (Millipore, Millicell ERS-2 Voltohmmeter, Billerica, MA); stable TEER values of approximately 1200 Ω was indicative of an intact monolayer. The terms apical and basolateral were used to describe the upper and lower chambers of cell inserts. Only those cells that successfully formed a monolayer were used. Two cellular systems were used to evaluate conditions related to pancreas and liver. iNS-1E cells were seeded on the basolateral side of the system at a density of 1 x 10^5^ cells /cm^2^. HepG2 were seeded at a density of 6 x 10^3^ cells/cm^2^.

### Glucose transport in epithelial cells

To evaluate the effect of the ANC present in purple corn pericarp on intestinal glucose transport, Caco-2 cells were differentiated in a monolayer as described in the cell culture and dual-layered cell culture system section. The cells were treated with 1 to 100 μM of pure ANC (C3G, P3G, Pr3G, D3G, C3G-P, and CF-P), or with 0.125 mg/mL to 1 mg/mL of the anthocyanin-rich extract (PCW), along with 0.5 mM the glucose-analogue probe 2-Deoxy-2-[(7-nitro-2,1,3-benzoxadiazol-4-yl)amino]-D-glucose (2-NBDG) for 2 h. After treatment, the culture media from the basolateral side of the well was evaluated at an excitation/emission wavelength of 465/540 nm in black 96-well plates. The fluorescence intensity of the treatments was compared to the untreated control and was expressed as percentage of glucose transport compared to the control.

### Glucose-stimulated insulin secretion (GSIS)

Measurement of GSIS from iNS-1E cells was performed according to Asfari et al. [38]. iNS-1E cells were plated in the basolateral side of a dual-layered system as described in the cell culture and dual-layered cell culture system section. The cells were treated with 1 to 100 μM of pure ANC (C3G, P3G, Pr3G, D3G, C3G-P, and CF-P), with 0.125 mg/mL to 1 mg/mL of the anthocyanin-rich extract (PCW), or with 5 to 20 μM of a FFAR1 agonist (TAK-875). After 24 h, the media was replaced by RPMI 1640 without glucose, and the cells were incubated for 2 h to simulate starvation. The cells were then washed three times with modified Krebs-Ringer-bicarbonate-HEPES buffer (KRBH), containing 135 mM NaCl, 3.6 mM KCl, 0.5 mM NaH_2_PO_4_, 0.5 mM MgCl_2_, 10 mM HEPES, 2 mM NaHCO_3_, and 1.5 mM CaCl_2_. Cells were incubated in 0 mM glucose modified KRBH for 30 min. The KRBH was replaced by KRBH containing 0.1% bovine serum albumin (BSA) and 20 mM glucose for stimulation. The cells were then incubated for another 30 min. The 0 mM glucose KRBH treated wells were used as negative control. The medium was collected and cleared by centrifugation at 15,000 x *g* for 1 min and stored at −20°C until assay using an insulin ELISA kit (ThermoFisher, Lafayette, CO).

### Glucose uptake

HepG2 cells were prepared in dual-layered system as indicated in the cell culture and dual-layered cell culture system section. Once independent cultures were ready to use, Caco-2 inserts were placed over HepG2 cultured on black 96-well plates. HepG2 cells were cultured overnight in low-glucose serum-free DMEM (5.5 mM) supplemented with 0.25% bovine serum albumin (BSA). On the day of the experiment, Caco-2 cells were apically treated with 100 μM of pure ANC (C3G, P3G, Pr3G, D3G, C3G-P, and CF-P) or 0.5 mg/mL of the anthocyanin-rich extract (PCW) for 24h. After treatment, Caco-2 cells were removed and media from HepG2 cells was changed to DMEM supplemented with 0.5 mM 2-DNBG and incubated for 4 h. Subsequently, the cell monolayer was evaluated for fluorescence intensity. Cells were lysed using RIPA Lysis Buffer System (Santa Cruz Biotechnology, Santa Cruz, CA) and assayed for protein concentration through the DC protein assay (Bio-Rad, Richmond, CA) with BSA as standard. Results were expressed as the percentage of glucose uptake compared to the untreated cells.

### Protein immunoblotting

The effect of the ANC from purple corn on selected proteins expression was assessed by western blot. iNS-1E and HepG2 cells were cultured in dual-layered system as described in the cell culture and dual-layered cell culture system section. The cells were seeded in the basolateral side of the system in 6-well plates. On the day of the experiment, Caco-2 cells were apically treated with 100 μM of pure ANC (C3G, P3G, Pr3G, D3G, C3G-P, and CF-P) or 0.5 mg/mL of the anthocyanin-rich extract (PCW) for 24h. Subsequently, the cells were lysed with RIPA buffer; lysates were separated through SDS-PAGE and transferred to PVDF Hybond-P membranes; primary and secondary (GE Healthcare, Buckinghamshire, UK) antibodies were used following manufacturer’s recommended dilutions for western blot. Protein expression was detected using 1:1 chemiluminescent reagents of ECL Prime Western Blotting kit (GE Healthcare, Buckinghamshire, UK) and visualized using a Gel Logic 4000 Pro Imaging System. After ECL detection, the membranes were washed, stripped with Restore PLUS Western Blot stripping buffer (Thermo Scientific, Lafayette, CO), re-blocked, and re-probed with GAPDH antibody. The intensity of each band was normalized to GAPDH (sc-47724), and the results were expressed as expression level relative to a control.

### Indirect measurement of free fatty acid receptor 1 activity

The activity of FFAR1 was assessed indirectly through the quantification of intracellular inositol monophosphate (IP) using an IP-One ELISA kit (Cisbio, Bedford, MA) following the manufacturer’s instructions. Briefly, iNS-1E cells were seeded in dual-layered system as described in the cell culture and dual-layered cell culture system section in 96-well plates. On the day of the experiment, the cells were incubated at 37°C for 1 h with stimulation buffer (146 mM NaCl, 4.2 mM KCl, 0.5 mM MgCl2, 1 mM CaCl2, 10 mM HEPES, 5.5 mM glucose, and 50 mM LiCl, pH 7.4). Concomitantly, the Caco-2 cells were treated apically with 1 to 300 μM of pure ANC (C3G, D3G, C3G-P, and CF-P), with 0.125 mg/mL to 0.5 mg/mL of the anthocyanin-rich extract (PCW), or with 1 to 100 μM of a FFAR1 agonist (TAK-875). The cells were lysed and transferred to an ELISA coated plate, and assayed for IP detection at 450 nm.

### Glucokinase activity

The ability of ANC from purple corn to activate glucokinase was assessed using the method of Dhanesha et al. [39]. HepG2 cells were prepared in dual-layered system as indicated in the cell culture and dual-layered cell culture system section in 6-well plates. The cells were treated apically with 1 to 300 μM of pure ANC (C3G, D3G, C3G-P, and CF-P), with 0.125 mg/mL to 0.5 mg/mL of the anthocyanin-rich extract (PCW), or with 1 to 100 μM of a GK synthetic agonist (Ro-28-1675). The cells were homogenized in a buffer containing 20 mM K_2_HPO_4_, 1 mM EDTA, 110 mM KCl, and 5 mM dithiothreitol (pH 7.4). The homogenates were lysed through sonication using 3 bursts of 3 seconds each. The lysates were cleared at 12,000 x *g* for 20 min at 4°C. A 100 U/mL of glucose-6 phosphate dehydrogenase enzyme solution was added to a reaction cocktail containing glucose, 60 mM Tris-HCl buffer (pH 9), 20 mM MgCl2, 4 mM ATP and 0.9 mM NADP. The reaction was equilibrated for 5 min, and 20 μg of protein/mL of the cell lysates were added. The absorbance was monitored at 320 nm in kinetic mode for 10 min. Correction of hexokinase activity was assessed by subtracting the activity measured with 0.5 mM glucose (measures only low Km hexokinases) from that activity obtained at 360 mM glucose (measures all hexokinases, including glucokinase).

### Confocal immunofluorescence microscopy

iNS-1E and HepG2 cells were seeded on 8-well μ-slide cultivation chambers for immunofluorescence staining (Ibidi, Am Klopferspitz, Germany) at a density of 2 x 10^4^ viable cells/well. The cells were treated with with 100 μM of pure ANC (C3G, P3G, Pr3G, D3G, C3G-P, and CF-P) or 0.5 mg/mL of the anthocyanin-rich extracts (PCW) for 24h. At the end of treatment, cells were fixed with 4% paraformaldehyde (Electron Microscopy Sciences, Hatfield, PA), permeabilized with 0.1% Triton X-100, and blocked with Image-iT FX Signal Enhancer (Invitrogen, Carlsbad, CA). The cells were incubated overnight with specific antibodies for FFAR1 or GK. Cells were then washed and stained with secondary antibodies coupled with a fluorescent probe for 3 h avoiding light exposure. Subsequently, cells were covered with ProLong Gold Antifade Mountant with 4′,6-Diamidine-2′-phenylindole dihydrochloride (DAPI) (Molecular Probes, Eugene, OR) for 24h at room temperature, avoiding light exposure. The chambers were stored at 4°C until further use. The fluorescence intensity was determined with a Zeiss LSM 880 laser-scanning confocal microscope (Carl Zeiss AG, Germany) and images captured with a 63×/1.4 Oil DIC M27 objective. The individual channels were obtained using a sequential scanning mode to prevent bleed-through of the excitation signal. Laser power, gain, and offset were kept constant across the samples, scanned in a high-resolution format of 1024 × 1024 pixels averaging four frames. Single optical planes of the individual channels were captured and all of the optical planes were displayed as a gallery. Individual images were analyzed for mean intensities and areas at multiple regions of interest among several cells in the field of view and expressed as average relative intensity in arbitrary units (AU) per area of analysis (μm^2^) in the membrane area of the cells using the program AxioVision Rel 4.8 (Carl Zeiss, Jena, Germany). All of the image panels were resized and consolidated. The brightness of the final collage displayed was then increased by 20% as a whole.

### Statistical analysis

All the experiments were independently conducted in, at least, duplicate. Data were analyzed by one-way ANOVA and *post hoc* Tukey’s test for multiple mean comparison, and Dunnet’s test for analysis where samples were compared to a control. Differences were considered significant at p < 0.05. Statistical analyses were performed using software JMP V 7.0 (SAS Institute, Cary, NC).

## Results and discussion

### Semi purification and identification of condensed forms

**Fig 1:**
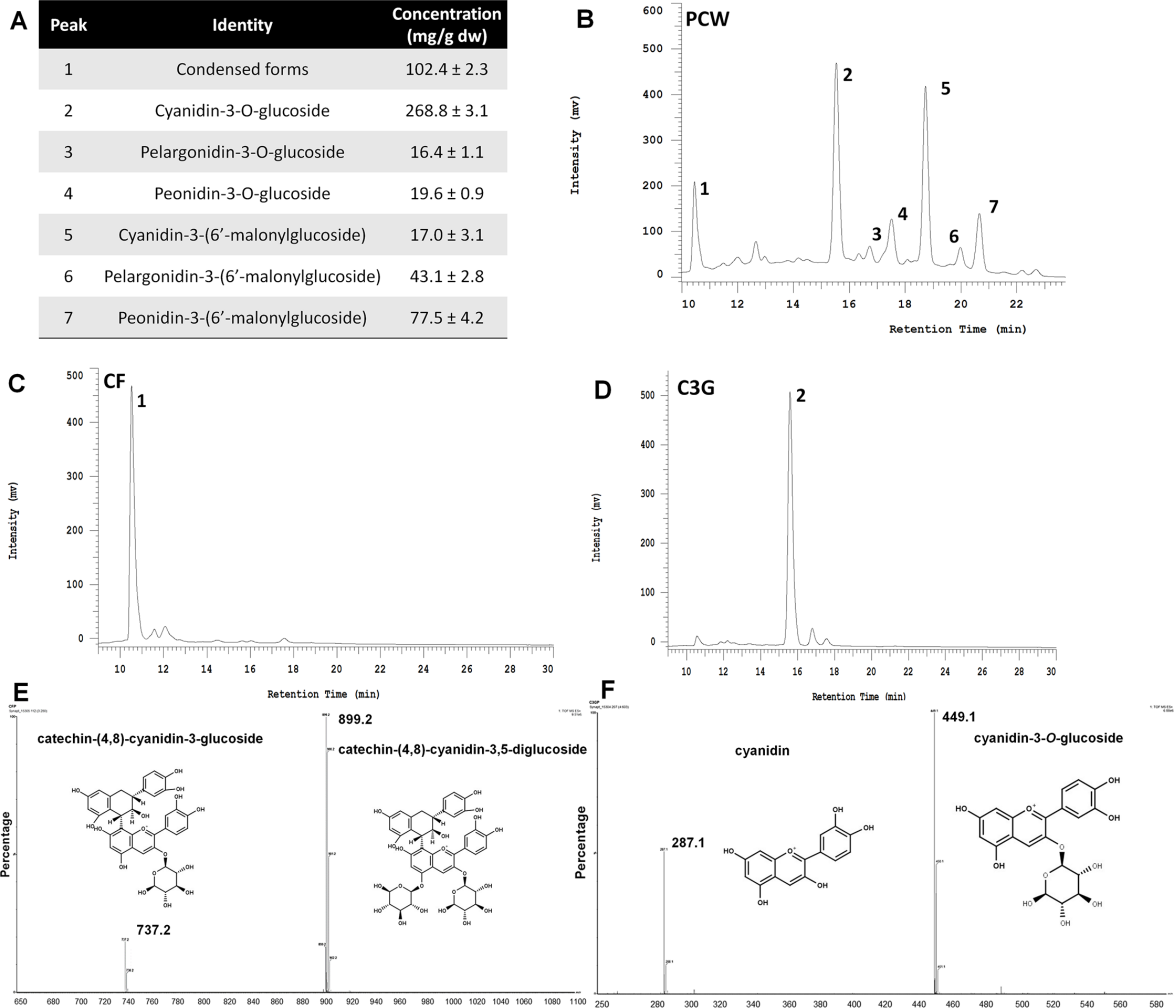
Characterization and isolation of anthocyanins present in the purple corn pericarp water extract. A) Quantification of anthocyanins present in PCW. The results are expressed as the mean ± SD of three independent determinations. B) Representative chromatogram of purple corn pericarp water extract (PCW) at 520 nm showing the different anthocyanins (ANC) identified as follows: 1 –condensed form of catechin-(4,8)-cyanidin-3,5-diglucoside; 2 –cyanidin-3-*O*-glucoside; 3—pelargonidin-3-*O*-glucoside; 4 –peonidin-3-*O*-glucoside; 5 –cyanidin-3-(6’-malonylglucoside); 6—pelargonidin-3-(6’-malonylglucoside); 7—peonidin-3-(6’-malonylglucoside). HPLC chromatogram of C) the semi-purified condensed form and D) semi-purified cyanidin-3-*O*-glucoside, isolated from PCW. Representative MS^2^ spectra of E) the semi-purified condensed form, and F) the semi-purified cyanidin-3-*O*-glucoside, showing the fragmentation pattern and structures of the compounds isolated from PCW.

**Fig 2:**
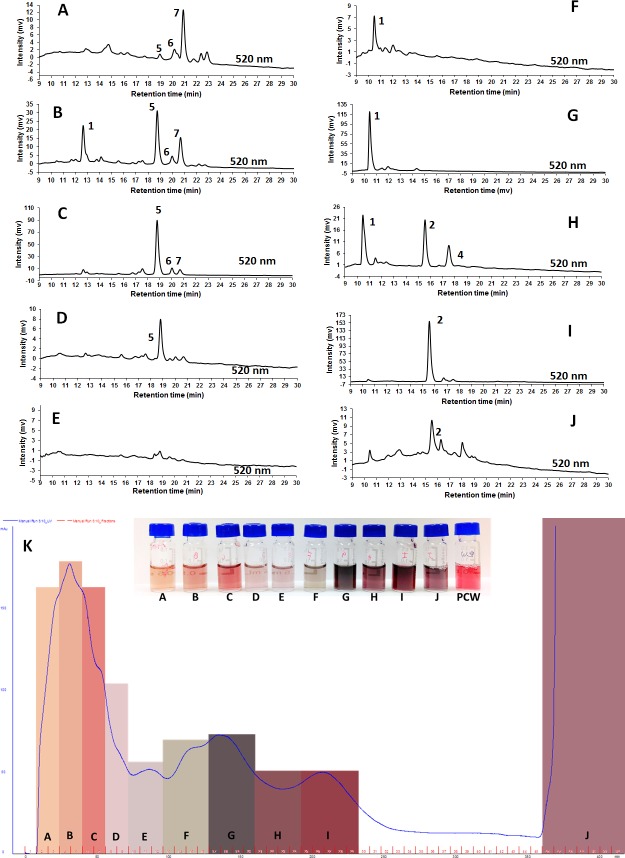
Fractionation of the purple corn pericarp water extract. A-J) Chromatograms from HPLC identification of the different fractions obtained from the purple corn pericarp water extract (PCW). K) Preparative phase liquid chromatography output at 280 nm showing the different fractions collected. The anthocyanins (ANC) were identified as follows: 1 –condensed form of catechin-(4,8)-cyanidin-3,5-diglucoside; 2 –cyanidin-3-*O*-glucoside; 3—pelargonidin-3-*O*-glucoside; 4 –peonidin-3-*O*-glucoside; 5 –cyanidin-3-(6’-malonylglucoside); 6—pelargonidin-3-(6’-malonylglucoside); 7—peonidin-3-(6’-malonylglucoside). The chromatograms shown in this figure were used exclusively for ANC identification purposes.

The concentration of ANC present in PCW is shown in **Fig 1A,** being C3G the major ANC. A representative chromatogram at 520 nm is shown in **Fig 1B**. After passing PCW through an Amberlite® column, it was possible to separate some individual ANC in different fractions (**Fig 2A–2J**) based in a preparative phase liquid chromatography output (**Fig 2K**). The fractions that contained C3G (**Fig 1C**) and the CF (**Fig 1D**) were isolated, and after passing them again through the column, a purity of 90% was achieved. From the tandem-mass spectrometry output, the C3G identity (**Fig 1E**) was confirmed since its fragmentation pattern agreed with previously reported spectra [40]. Regarding CF-P, the MS^2^ spectra showed two main fragments with an m/z of 737.2 and 899.2 (**Fig 1F**). This fragmentation pattern concurs with that found in colored corn by Gonzalez-Manzano et al., [41] for catechin-(4,8)-cyanidin-3,5-diglucoside.

### Anthocyanins from purple corn reduced epithelial glucose transport

**Fig 3:**
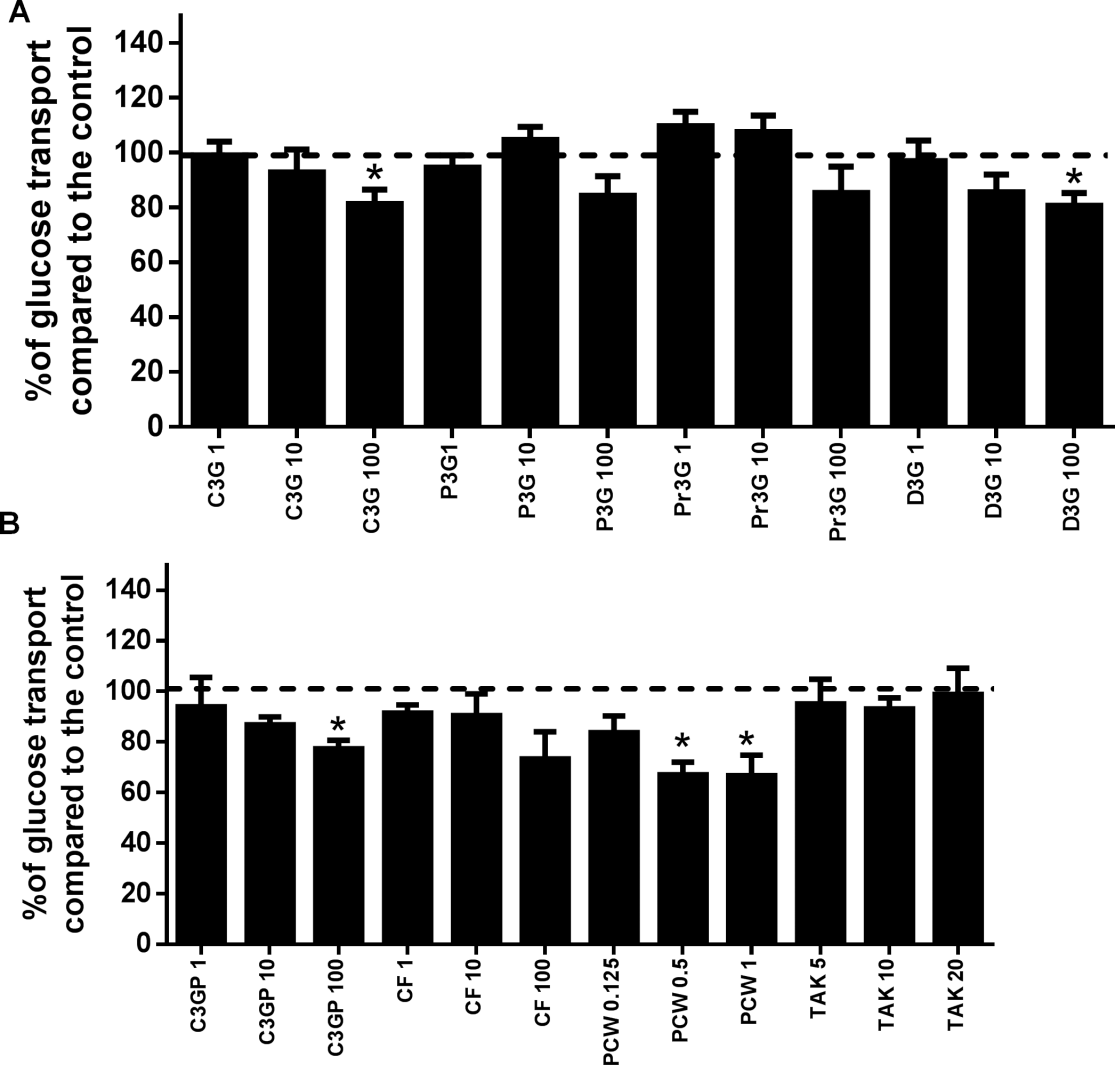
Glucose transport in Caco-2 cells in response to treatment with anthocyanins from purple corn. Modulation of glucose transport in Caco-2 cells as a response to A) cyanidin-3-*O*-glucoside (C3G), peonidin-3-*O*-glucoside (P3G), pelargonidin-3-*O*-glucoside (Pr3G), delphinidin-3-*O*-glucoside (D3G); and B) the semipurified C3G from purple corn pericarp (C3GP) catechin-(4,8)-cyanidin-3,5-diglucoside condensed form (CF), and purple corn pericarp water extract (PCW). The treatments with pure anthocyanins (ANC) ranged from 1 μM to 100 μM, and PCW from 0.125 mg/mL to 1.0 mg/mL, the free fatty acid receptor-1 (FFAR1) agonist TAK-875 was tested from 5 μM to 20 μM. Results are expressed as the mean ± SD of three independent experiments. A dotted line representing 100% was added for reference. Bars with a star means statistical difference (p < 0.05) compared to untreated cells as determined by Dunnet’s test.

In this experiment, we used ANC found in purple corn. For comparative purposes, we also included D3G throughout all the experiments, and even though it is not present in corn, previous reports have shown a greater bioactivity compared with other ANC [42, 43]. The results of glucose transport in Caco-2 cells are shown in **Fig 3**. Only the highest concentration of the pure ANC (100 μM) was efficient at reducing glucose transport in the epithelial cells ranging from 15% (C3G) (**Fig 3A**) to 27% (CF-P) (**Fig 3B**) reduction. PCW reduced roughly 33% of glucose transport at a concentration of 0.5 mg/mL. Our results agree with some studies conducted to evaluate the potential of ANC from other sources, which were found to reduce glucose transport in Caco-2 cells; namely, apple, strawberry, and grape’s polyphenolic extracts [44–46]. However, our results contrast with those found by Castro-Acosta et al. [44], who used up to 600 μM of pure D3G and C3G and found no inhibition of the glucose transport in Caco-2 cells through detection of a D-[14C (U)] radioactive glucose as a tracer. This discrepancy could be due to different methodologies used. One of the main mechanisms described for the reduction in glucose transport by polyphenols, including ANC, is their interaction with the glucose transporter 2 (GLUT2) and the sodium-glucose linked transporter 1 (SGLT1)[47, 48]. A reduction in the glucose absorbed in the intestine is desirable in patients with hyperglycemia and type-2 diabetes, since high concentrations of blood sugar are associated with several known comorbidities [49].

### Anthocyanins from purple corn increased GSIS through modulation of FFAR1 in pancreatic β-cells

**Fig 4:**
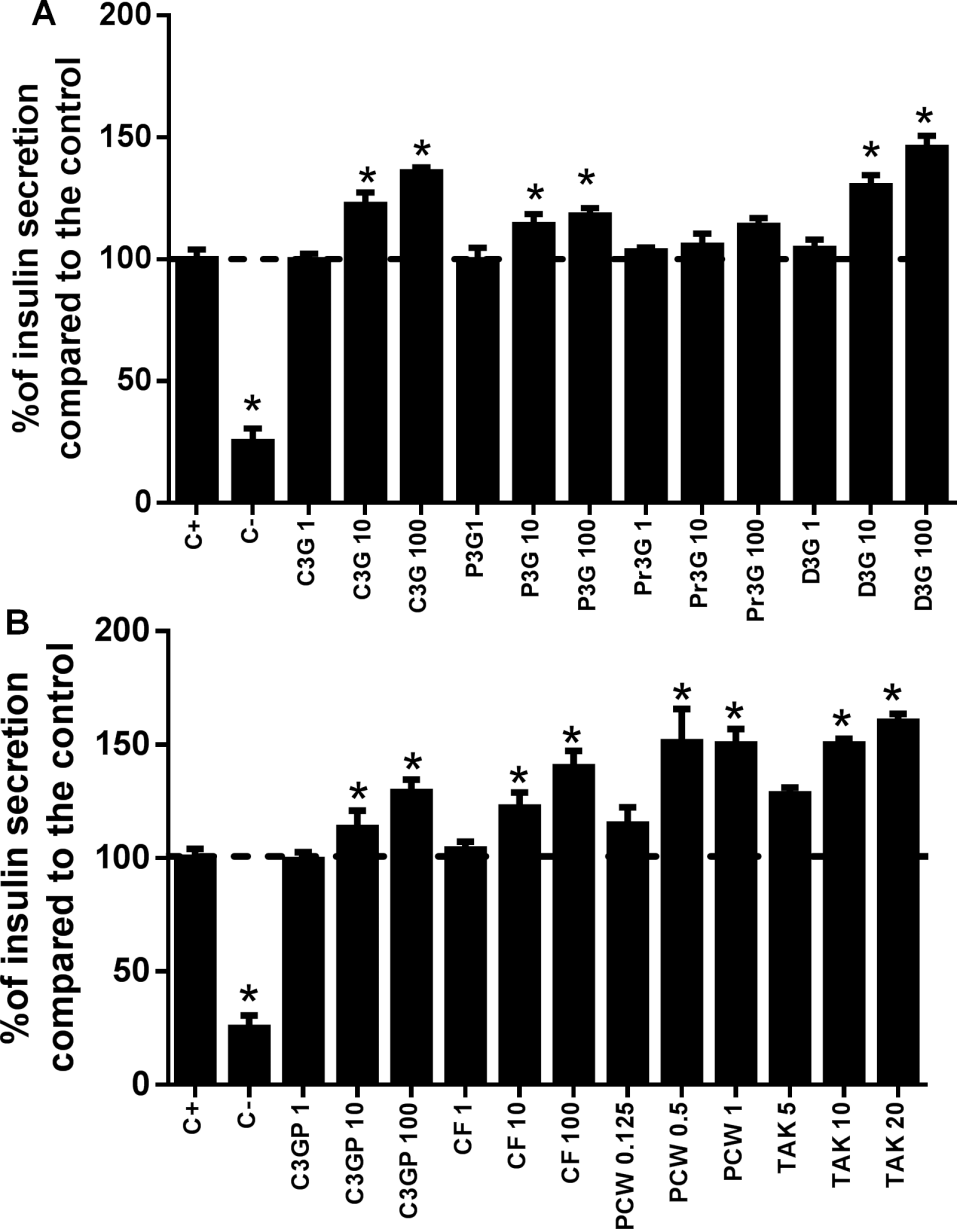
Effect of pure anthocyanins and anthocyanins-rich extracts from purple corn in glucose-stimulated insulin secretion in iNS-1E pancreatic β-cells. Modulation of insulin secretion in iNS-1E cells as a response to A) cyanidin-3-*O*-glucoside (C3G), peonidin-3-*O*-glucoside (P3G), pelargonidin-3-*O*-glucoside (Pr3G), delphinidin-3-*O*-glucoside (D3G); and B) the semipurified C3G from purple corn pericarp (C3GP) catechin-(4,8)-cyanidin-3,5-diglucoside condensed form (CF), purple corn pericarp water extract (PCW). The treatments with pure ANC were from 1 μM to 100 μM, and the anthocyanin-rich extracts from 0.125 mg/mL to 1.0 mg/mL, the free fatty acid receptor-1 (FFAR1) agonist TAK-875 was tested from 5 μM to 20 μM. The results are expressed as the mean ± SD of three independent experiments. A dotted line representing 100% was added for reference. Bars with a star means statistical difference (p < 0.05) compared to untreated cells as determined by Dunnet’s test.

The insulin secreting effect of ANC from purple corn is shown in **Fig 4A and 4B**. Among the pure ANC, the maximum insulin secreting effect was observed for D3G at 100 μM (45% higher than the control), followed by 100 μM CF-P (40% higher than the control). At 1 mg/mL, PCW increased insulin secretion by 52%. The synthetic FFAR1 agonist TAK-875, known to promote insulin exocytosis through FFAR1 stimulation, increased the insulin secretion of iNS-1E cells treated with 20 μM of the drug by 60%. Few reports are available about the increase in insulin secretion induced by ANC from food sources [50, 51]. A study from Suantawee et al. [52] reported that cyanidin (aglycone form of C3G) stimulated insulin secretion in iNS-1E cells through activation of ι-type voltage-dependent Ca^2+^ channels. However, ANC might have different mechanisms of action since it has been observed that anthocyanidins and ANC have different bioactivities [53].

**Fig 5:**
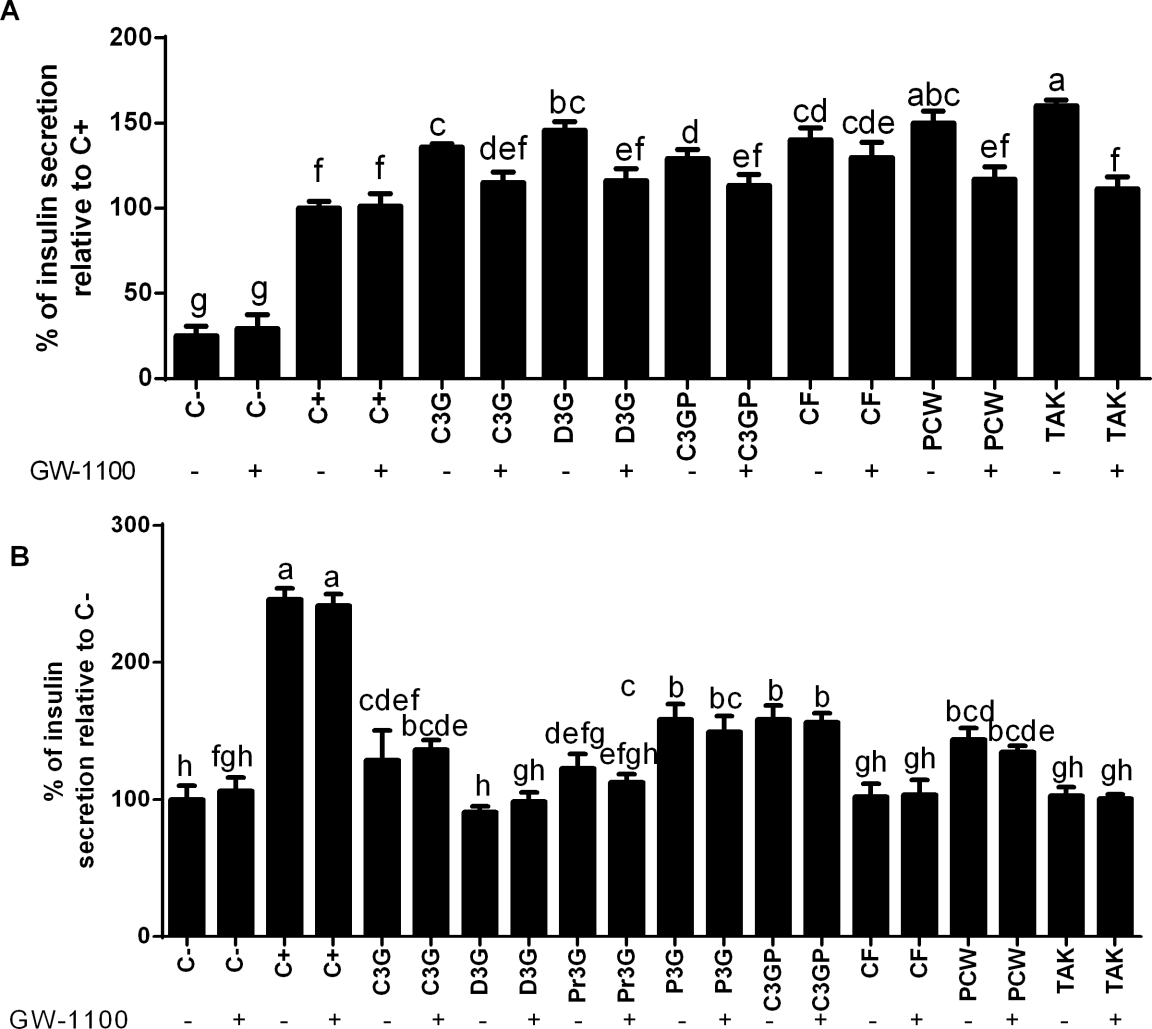
Effect of the anthocyanins from purple corn on insulin secretion with and without a FFAR1 inhibitor in pancreatic cells. A) Effect of the free fatty acid receptor-1 (FFAR1) antagonist GW-1100 in the insulin secreting activity of pure anthocyanins ANC and ANC-rich extracts from purple corn in iNS-1E pancreatic β-cells compared to positive control C+ (glucose). B) Insulin secreting activity of pure ANC and ANC-rich extracts from purple corn in glucose-starved pancreatic β-cells compared to negative control C- (starving conditions). The cells were treated with different concentrations of pure ANC (1 μM– 100 μM) cyanidin-3-*O*-glucoside (C3G), delphinidin-3-*O*-glucoside (D3G), pelargonidin-3-*O*-glucoside (Pr3G), peonidin-3-*O*-glucoside (P3G), the semipurified C3G from purple corn pericarp (C3GP), catechin-(4,8)-cyanidin-3,5-diglucoside condensed form (CF), the purple corn pericarp water extract (PCW) (0.125 mg/mL– 1.0 mg/mL), and a FFAR1 agonist TAK-875 (5 μM– 20 μM) in the presence or absence of 10 μM GW-1100. Results are expressed as the mean ± SD of three independent experiments. Bars with different letters means statistical difference (p < 0.05) as determined by Tukey’s test.

When the cells were treated with the ANC along with the specific FFAR1 antagonist GW-1100 (10 μM), their insulin secreting ability was significantly (p < 0.05) reduced (**Fig 5A**). These results suggest that the ability of ANC to enhance insulin secretion depends partially on the activation of FFAR1. To the best of our knowledge, this is the first report exploring the interaction of ANC with the novel diabetes marker FFAR1 as a potential mechanism to enhance insulin secretion in iNS-1E cells. In an independent experiment, glucose-starved iNS-1E cells were treated with the ANC, and with or without GW-1100 (**Fig 5B**). Two main outcomes were found. First, that C3G, P3G, C3G-P and PCW increased (p < 0.05) insulin secretion compared to the negative control up to 58% (50 μM C3GP). Therefore, they are potential candidates to act as insulin secretagogues. Additionally, it was found that there was no difference on the insulin secretion level when the cells were concomitantly treated with the ANC and the presence or absence of GW-1100, supporting the hypothesis that the insulinotropic FFAR1 activity is glucose-dependent [24]. The increase in insulin secretion is beneficial for patients with type-2 diabetes with relative insulin deficiency and insulin resistance [49]. From the results obtained at this stage, we eliminated P3G and Pr3G from the main studies since they did not show a significant effect in the iNS-1E cells.

**Fig 6:**
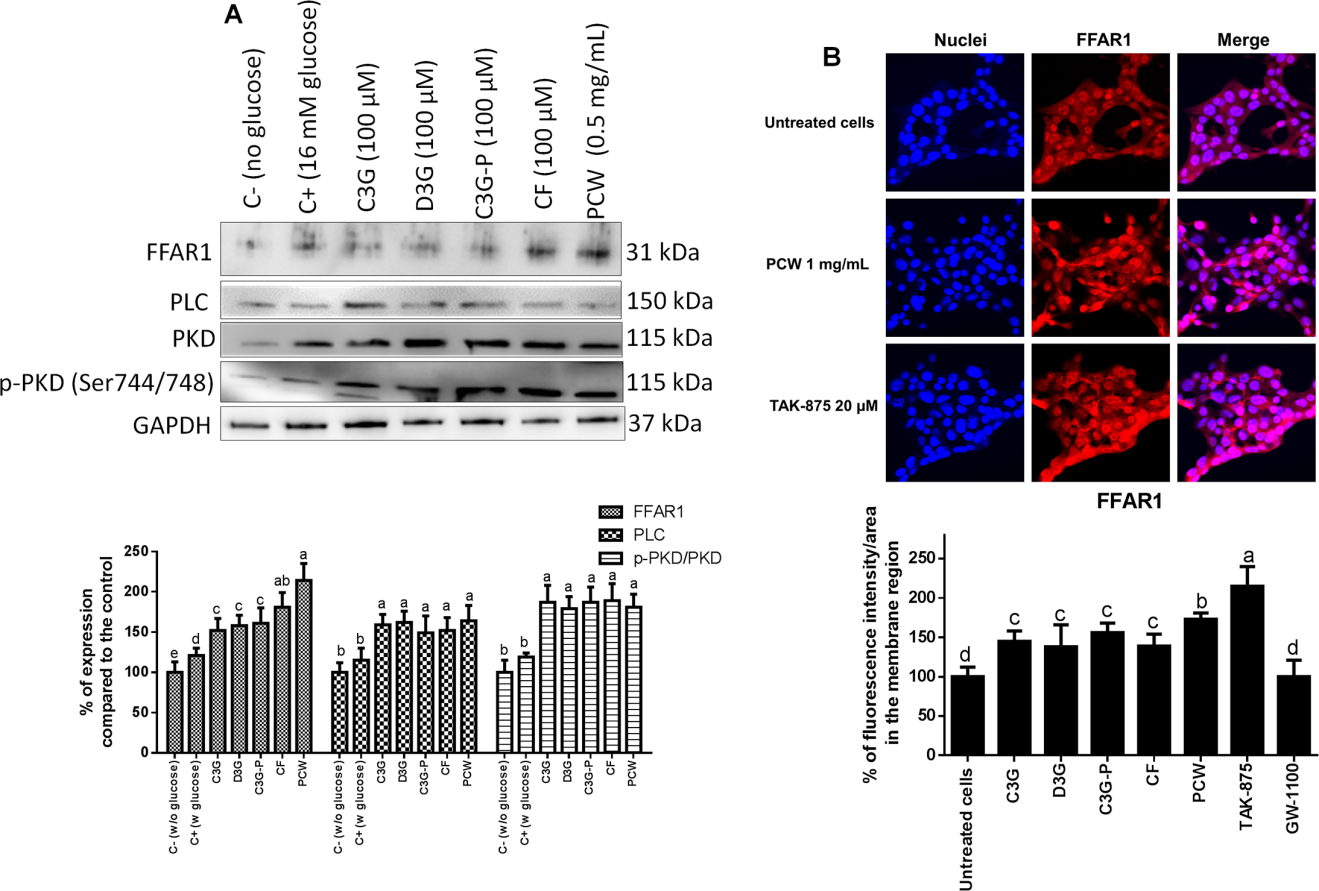
Effect of anthocyanins from purple corn on proteins related to FFAR1-dependent insulin secretion in iNS-1E cells. A) Effect of the pure anthocyanins (ANC) and ANC-rich extracts from purple corn on iNS-1E pancreatic β-cells on expression of proteins related to the free fatty acid receptor-1 (FFAR1)-dependent insulin secretory pathway. A representative image of the immunoblots is shown. Results are expressed as the mean ± SD of three independent experiments. Bars with different letters means statistical difference (p < 0.05) as determined by Tukey’s test. B) Confocal laser scanning microscopy depicting two-dimensional immunofluorescence localization of nuclei (blue) and FFAR1 (red). The quantification determined by the intensity (AU) over area (μm^2^) of FFAR1 is shown. Data represents the mean ± SE of four independent fields of view from two independent cellular replicates. Means with different letters at the same time point are significantly different (p < 0.05) according to Tukey’s test. The cells were treated with 100 μM of pure ANC cyanidin-3-*O*-glucoside (C3G), delphinidin-3-*O*-glucoside (D3G), the semipurified C3G from purple corn pericarp (C3GP), catechin-(4,8)-cyanidin-3,5-diglucoside condensed form (CF), and 0.5 mg/mL of the ANC-rich extracts from purple corn pericarp water extract (PCW), 20 μM of the FFAR1 agonist TAK-875, or 10 μM of FFAR1 antagonist GW-1100.

As determined by western blot (**Fig 6A**), the ANC from purple corn modulated the expression of proteins from the FFAR1-dependent insulin secretory pathway. PCW was the most efficient treatment, increasing (p < 0.05) the expression of FFAR1 by roughly 100% compared to the untreated control. C3G, D3G, C3G-P and CF-P increased (p < 0.05) the expression of FFAR1, however, in a lesser extent than the extracts. All treatments increased equally (p > 0.05) the expression of phospholipase C (PLC) by approximately 50% compared to the untreated control. The same phenomenon was observed for the phosphorylation of protein kinase D (PKD); all treatments increased its expression compared to the control by approximately 65%. To confirm the effect of ANC inducing FFAR1 expression, an immunofluorescence microscopy approach was used. The cellular immunolocalization of FFAR1 as a response to the treatment with ANC is shown in **Fig 6B**. TAK-875 increased (p < 0.05) the membrane expression of FFAR1 compared to the untreated control by 97%. PCW increased the membrane expression of FFAR1 by 61%, and no differences were found in expression of FFAR1 by C3G, D3G, C3G-P and CF-P, that increased the expression by roughly 45%. It has been proposed that FFAR1 is mainly coupled with the G protein α-subunit of the Gq family (Gαq) [54]. After stimulation of FFAR1, the α-subunit of receptor-associated heterotrimeric G protein G_q/11_ undergoes an activating GDP-for-GTP exchange and subsequently dissociates from the β/γ subunit. The GTP-bound α-subunit activates PLC promoting the cleavage of phosphatidyl inositol-4,5-bisphosphate (PIP2) located in the plasma membrane, and consequent generation of inositol 1,4,5-trisphosphate (IP3) and diacylglycerol (DAG). The latter can activate PKD which in turn phosphorylates and activates targets related to filamentous actin remodeling, potentiating the insulin granule exocytosis in pancreatic β-cells [55, 56].

**Fig 7:**
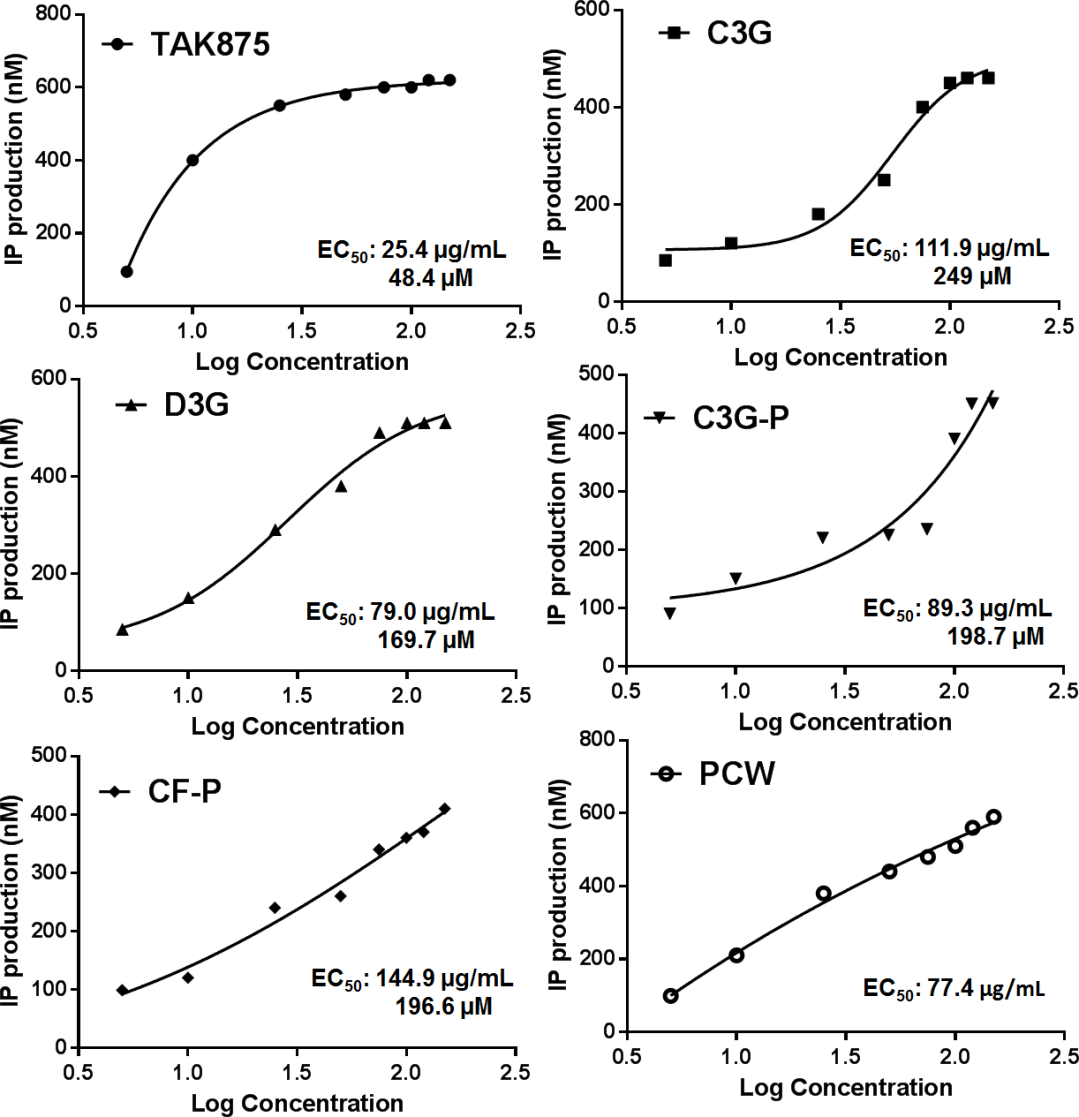
FFAR1-activating potential of pure anthocyanins and anthocyanins-rich extracts from purple corn determined indirectly through ELISA-based inositol monophosphate (IP) quantification. The cells were treated with a free fatty acid receptor-1 (FFAR1) agonist TAK-875 (5 μM– 20 μM), and different concentrations of pure anthocyanins (ANC): (1 μM– 300 μM) cyanidin-3-*O*-glucoside (C3G), delphinidin-3-*O*-glucoside (D3G), the semipurified C3G from purple corn pericarp (C3GP), catechin-(4,8)-cyanidin-3,5-diglucoside condensed form (CF), and the purple corn pericarp water extract (PCW) (0.125 mg/mL– 0.5 mg/mL), A non-linear regression was used to determine the half-maximal effective concentration (EC_50_).

To understand the potential of ANC to activate FFAR1, a biochemical assay was carried out to measure the indirect activation of the receptor. This was assessed by quantifying inositol phosphate (IP). It is known that stimulation of G protein-coupled receptors induces PLC activation and triggers the IP cascade. The results of IP quantification are shown in **Fig 7**. The lowest EC_50_ value was for the synthetic agonist TAK-875 (EC_50_ = 25.4 μg/mL). All the anthocyanin treatments showed to activate FFAR1 since they increased IP production. Among the treatments, PCW had the lowest EC_50_ (≈ 77 μg/mL). C3G was the most efficient FFAR1 activator (EC_50_ = 111.9 μg/mL) among the pure ANC. Previous reports have identified multiple ligand-binding sites for FFAR1[57]. Besides, cooperative allosteric regulation of FFAR1 has been reported [58]. This could partially explain the higher efficacy of the extracts compared to pure ANC to activate FFAR1, since different ANC present in PCWcould be binding to multiple sites.

### Anthocyanins from purple corn increased glucose uptake and activated GK in hepatic cells

**Fig 8:**
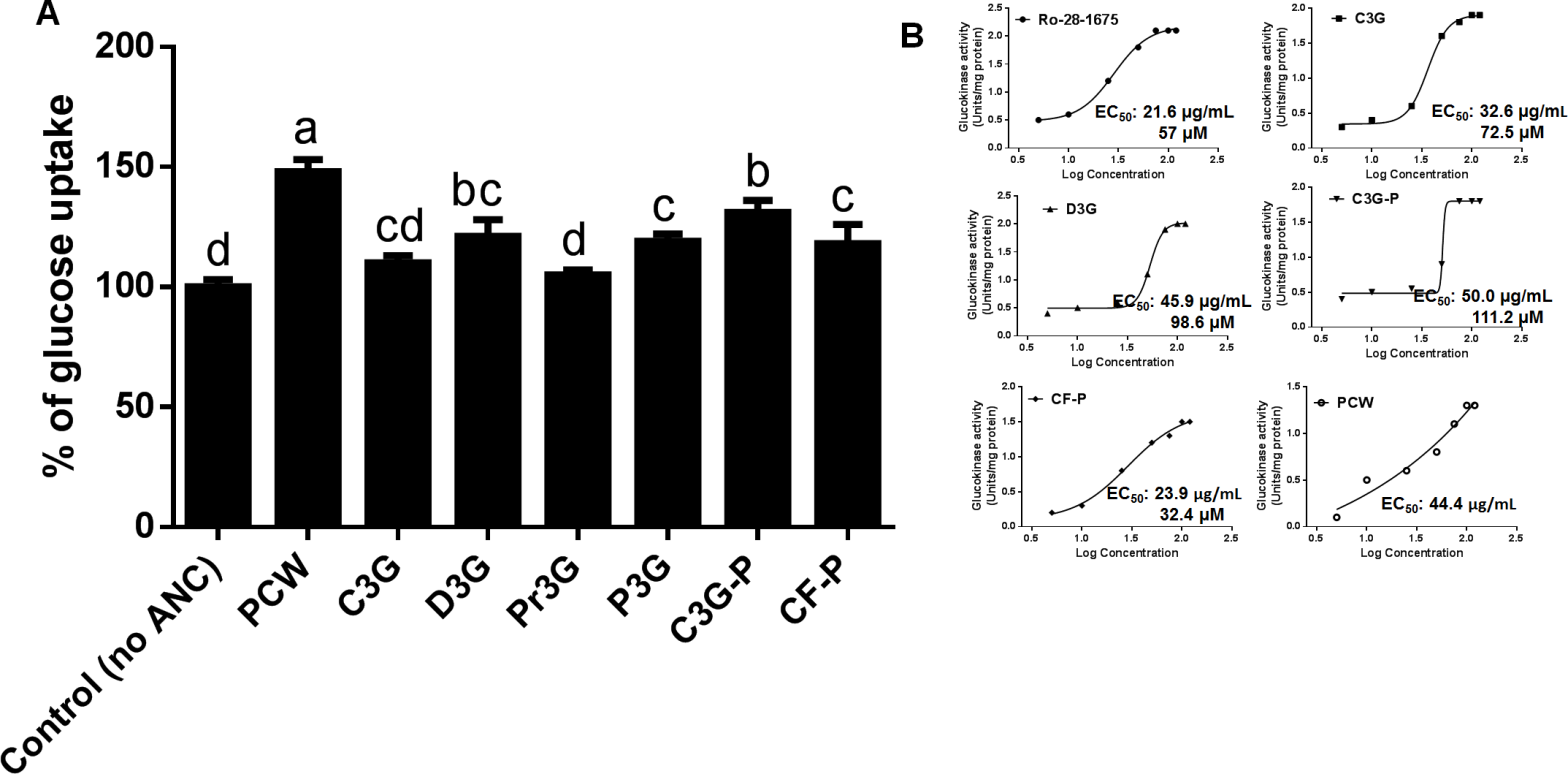
Effect of anthocyanins from purple corn on glucose uptake and GK-activating potential in HepG2 cells. A) Effect of pure anthocyanins (ANC) and ANC-rich extracts from purple corn in glucose uptake in HepG2 cells. The cells were treated with 0.4 mg/mL of the ANC-rich extract from purple corn pericarp water extract (PCW), 50 μM of pure ANC cyanidin-3-*O*-glucoside (C3G), delphinidin-3-*O*-glucoside (D3G), pelargonidin-3-*O*-glucoside (Pr3G), peonidin-3-*O*-glucoside (P3G), the semipurified C3G from purple corn pericarp (C3GP) and catechin-(4,8)-cyanidin-3,5-diglucoside condensed form (CF). Results are expressed as the mean ± SD of three independent experiments. Bars with different letters means statistical difference (p < 0.05) as determined by Tukey’s test. B) GK activation by ANC from purple corn as determined using a biochemical assay. A non-linear regression was used to determine the half-maximal effective concentration (EC_50_) based on the method by Sebaugh [61]. The cells were treated with a glucokinase agonist Ro-28-1675 (1 μM– 100 μM), different concentrations of pure ANC (1 μM– 300 μM), and PCW (0.125 mg/mL– 0.5 mg/mL). The results are expressed as units of glucokinase activity per milligram of protein in the cell lysates (Units/mg protein) versus the log concentration of the samples.

The glucose uptake by HepG2 hepatic cells in response to ANC from purple corn is shown in **Fig 8A**. All the treatments, except for Pr3G, increased (p < 0.05) the glucose uptake in hepatic HepG2 cells. PCW was the most effective treatment increasing by approximately 45% the glucose uptake. From the pure ANC, D3G and C3G-P were the most effective increasing the glucose uptake (35% and 41%, respectively). An increase in hepatic glucose uptake contributes to maintain glucose homeostasis in hyperglycemic conditions [59]. Additionally, the effect on the enzymatic activity of GK was assessed through a biochemical assay. As can be observed in **Fig 8B**, CF-P had the greatest activation effect on GK, having an EC_50_ of 23.9 μg/mL, followed by PCW, with EC_50_ of 44.4 μg/mL. In terms of GK activity expressed as Units/mg of protein, D3G was the most effective treatment reaching 2.1 Units/mg protein at the highest concentration. GK is the rate-limiting enzyme that transforms glucose to glucose-6-phosphate during glycolysis [60]. GK activity and expression is reduced in the liver of diabetic patients, therefore it is desirable to promote its activity [27].

**Fig 9:**
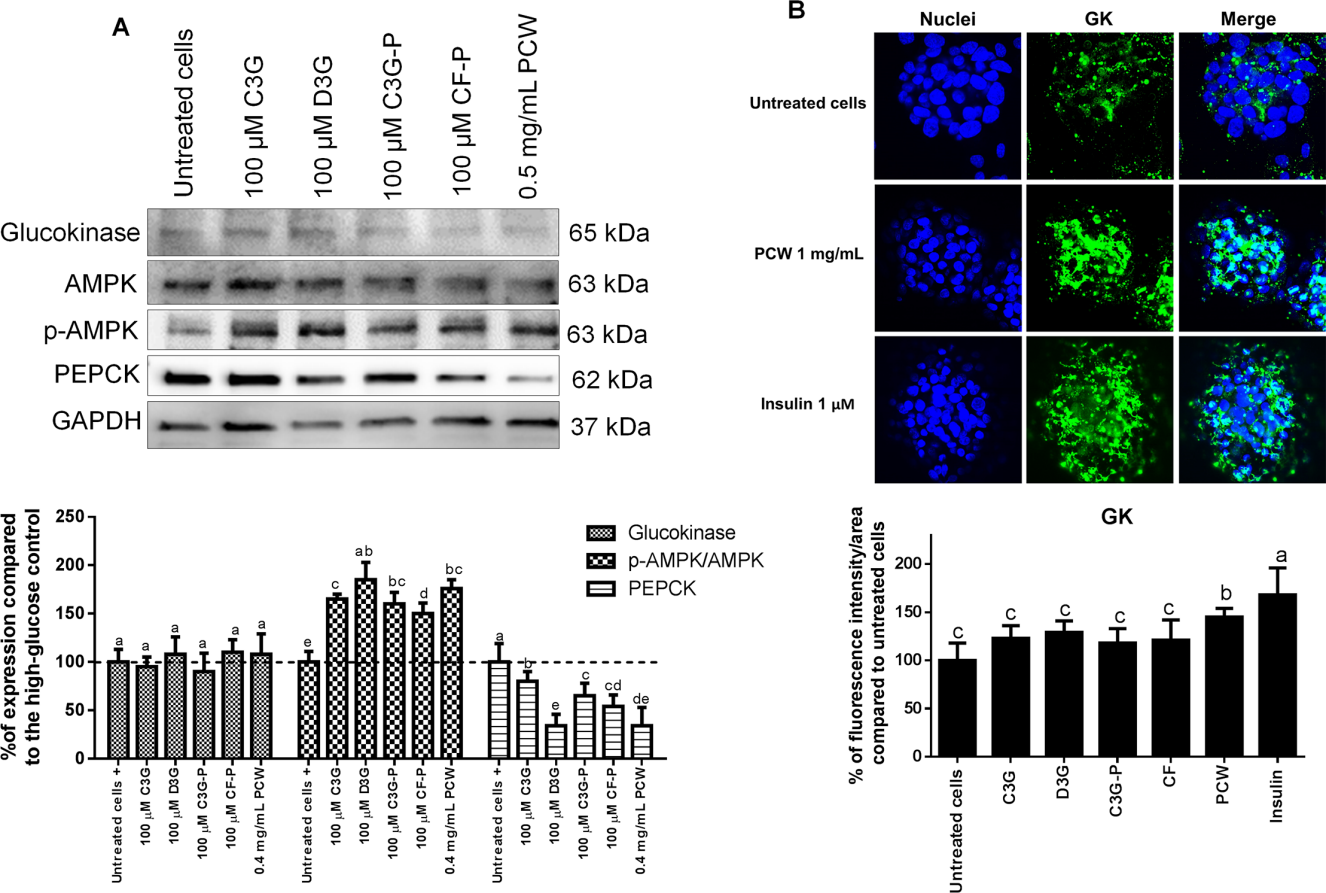
Effect of anthocyanins from purple corn on proteins related to glucose metabolism in HepG2 cells. A) Effect of the pure anthocyanins (ANC) and ANC-rich extracts from purple corn on HepG2 hepatic cells on expression of proteins related to glucose metabolism. A representative image of the immunoblots is shown. Results are expressed as the mean ± SD of three independent experiments. Bars with different letters means statistical difference (p < 0.05) as determined by Tukey’s test. B) Confocal laser scanning microscopy depicting two-dimensional immunofluorescence localization of nuclei (blue) and GK (green). The quantification determined by the intensity (AU) over area (μm^2^) of free fatty acid receptor-1 (FFAR1) is shown. Data represents the mean ± SE of four independent fields of view from two independent cellular replicates. Means with different letters at the same time point are significantly different (p < 0.05) according to Tukey’s test. The cells were treated with 100 μM of pure ANC cyanidin-3-*O*-glucoside (C3G), delphinidin-3-*O*-glucoside (D3G), semipurified C3G from purple corn pericarp (C3GP), catechin-(4,8)-cyanidin-3,5-diglucoside condensed form (CF), and 0.5 mg/mL of the ANC-rich extracts from purple corn pericarp water extract (PCW) in comparison to just insulin (1 μM).

The ANC present in purple corn modulated proteins related to the carbohydrate metabolism of HepG2 cells (**Fig 9A)**. The expression of GK remained constant throughout all the treatments compared to the untreated control (p > 0.05). However, all the treatments increased the phosphorylation of AMP-activated protein kinase (AMPK) in different levels ranging from 48% to 89% compared with the untreated cells; D3G and PCWwere the most effective treatments. Correlating with AMPK phosphorylation, the ANC reduced the expression of phosphoenolpyruvate carboxykinase (PEPCK). Consistently, D3G and PCWwere the most potent treatments reducing PEPCK levels. AMPK is an intracellular energy sensor involved in the maintenance of cellular metabolism and its activation is known to suppress gluconeogenesis in the liver [62]. Active AMPK phosphorylates glycogen synthase kinase 3-β (GSK3β), which suppresses glucose-6-phosphatase (G6Pase) and PEPCK (major enzyme responsible for the regulation of gluconeogenesis), and consequently decreases hepatic glucose production [63], contributing to reduced levels of circulating blood sugar. Contrasting with the immunoblotting results, when the cells were evaluated through confocal microscopy, an increase in GK expression by 46% in HepG2 cells by PCW was observed, and no differences were found among the other treatments and the untreated cells (**Fig 9B**). The differences in the two methods could be associated to the high sensitivity of the immunocytochemical protocol.

## Conclusions

**Fig 10:**
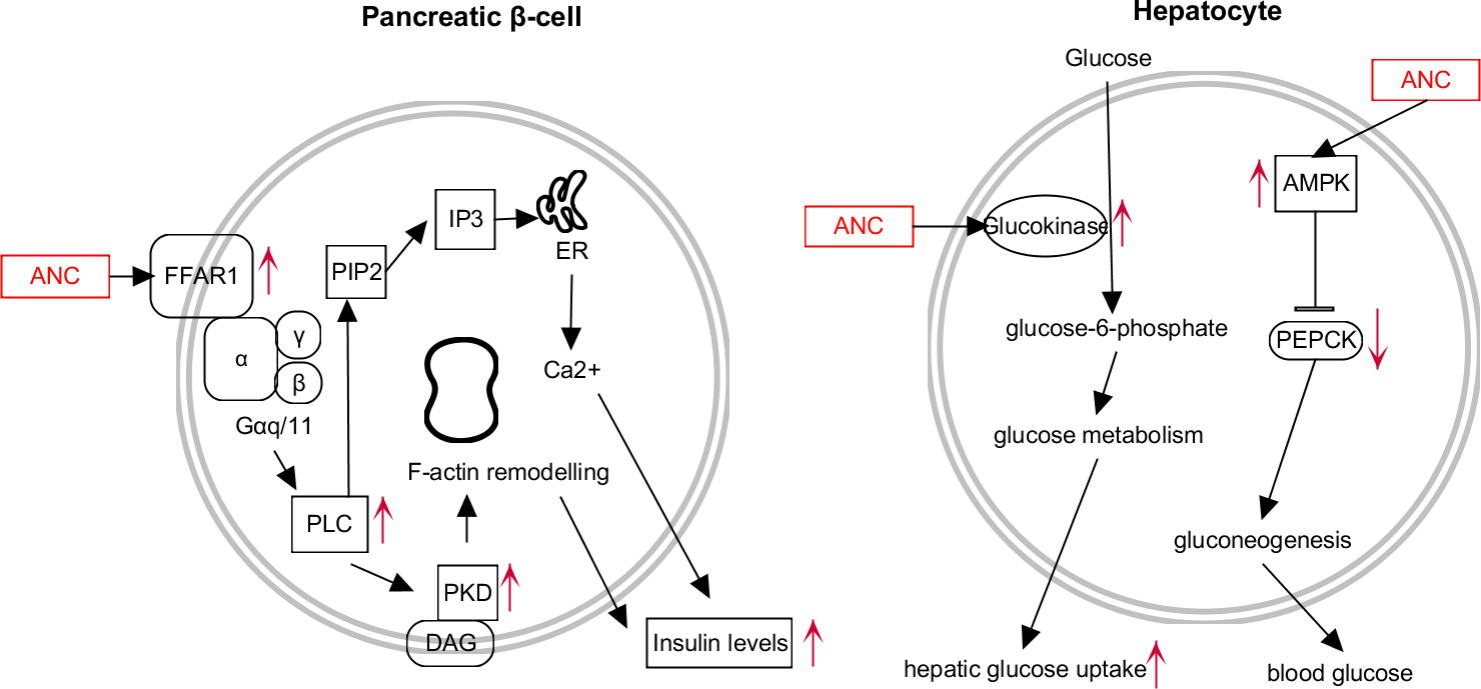
Summary of the effects of the anthocyanins from purple corn on the free fatty acid receptor-1 (FFAR1)-dependent effects on pancreatic β-cells, and in glucose metabolism of hepatic cells. The anthocyanins from purple corn activated FFAR1 in iNS-1E pancreatic cells and increased the glucose-dependent insulin secretion. In HepG2 cells, the anthocyanins activated glucokinase activity and promoted the phosphorylation of AMPK. Markers with an up- or down-arrow indicate modulation in response to the ANC of purple corn.

The findings of this study open the possibility to use ANC from purple corn as candidates to activate FFAR1 and GK, known markers that when activated ameliorate type-2 diabetes and its complications. For the first time, we report that ANC in purple corn increase insulin secretion in pancreatic β-cells *in vitro* potentially through activation of FFAR1. A summary of the effects of the ANC from purple corn in pancreatic β-cells and hepatocytes is shown in **Fig 10.** The implications of this discovery provide basic understanding to propose future studies, such as *in vivo* research to confirm this bioactivity, and validation of possible allosteric cooperation in the activation of FFAR1 between ANC and drugs. In addition, the ANC from purple corn enhanced *in vitro* hepatic glucose uptake, which can contribute to maintaining glucose homeostasis. In general, the anthocyanin-rich extracts were more effective than pure ANC. The results of this study suggest that ANC from purple corn are good candidates to be incorporated in the diet during type-2 diabetes treatment.
